# Plasmon-activated water as a therapeutic strategy in Alzheimer’s disease by altering gut microbiota

**DOI:** 10.18632/aging.204706

**Published:** 2023-05-08

**Authors:** Chia-Hsiung Cheng, Yu-Chuan Liu, Yu-Chen S.H. Yang, Kun-Ju Lin, Dean Wu, Yun-Ru Liu, Chun-Chao Chang, Chien-Tai Hong, Chaur-Jong Hu

**Affiliations:** 1 Department of Biochemistry and Molecular Cell Biology, School of Medicine, College of Medicine, Taipei Medical University, Taipei 11031, Taiwan; 2 Cell Physiology and Molecular Image Research Center, Wan Fang Hospital, Taipei Medical University, Taipei 11031, Taiwan; 3 Joint Biobank, Office of Human Research, Taipei Medical University, Taipei 11031, Taiwan; 4 Department of Nuclear Medicine and Molecular Imaging Center, Linkou Chang Gung Memorial Hospital, Taoyuan City 33302, Taiwan; 5 Healthy Aging Research Center and Department of Medical Imaging and Radiological Sciences, College of Medicine, Chang Gung University, Taoyuan City 33302, Taiwan; 6 Department of Neurology, Shuang Ho Hospital, Taipei Medical University, New Taipei City 23561, Taiwan; 7 Department of Neurology, School of Medicine, College of Medicine, Taipei Medical University, Taipei 11031, Taiwan; 8 Sleep Center, Shuang Ho Hospital, Taipei Medical University, New Taipei City 23561, Taiwan; 9 Division of Gastroenterology and Hepatology, Department of Internal Medicine, Taipei Medical University Hospital, Taipei 11031, Taiwan; 10 Division of Gastroenterology and Hepatology, Department of Internal Medicine, School of Medicine, College of Medicine, Taipei Medical University, Taipei 11031, Taiwan; 11 TMU Research Center for Digestive Medicine, Taipei Medical University, Taipei 11031, Taiwan; 12 Department of Neurology and Dementia Center, Shuang Ho Hospital, Taipei Medical University, New Taipei City 23561, Taiwan; 13 PhD program of Medical Neuroscience, College of Medical Science and Technology, Taipei Medical University, Taipei 11031, Taiwan

**Keywords:** Alzheimer’s disease, plasmon-activated water, fecal microbiota transplantation, microbiota, beta amyloid, tau

## Abstract

Gut microbiota (GM) are involved in the pathophysiology of Alzheimer’s disease (AD) and might correlate to the machinery of the gut-brain axis. Alteration of the GM profiles becomes a potential therapy strategy in AD. Here, we found that plasmon-activated water (PAW) therapy altered GM profile and reduced AD symptoms in APPswe/PS1dE9 transgenic mice (AD mice). GM profile showed the difference between AD and WT mice. PAW therapy in AD mice altered GM profile and fecal microbiota transplantation (FMT) reproduced GM profile in AD mice. PAW therapy and FMT in AD mice reduced cognitive decline and amyloid accumulation by novel object recognition (NOR) test and amyloid PET imaging. Immunofluorescent staining and western blot analysis of β-amyloid (Aβ) and phosphorylated (p)-tau in the brain of AD mice were reduced in PAW therapy and FMT. The inflammatory markers, interleukin (IL)-6, IL-1β, and tumor necrosis factor (TNF)-α and pro-inflammatory indicator of arginase-1/CD86 ratio were also reduced. Furthermore, immunohistochemistry (IHC) analysis of occludin and claudin-5 in the intestine and AXL in the brain were increased to correlate with the abundant GM in PAW therapy and FMT. Our results showed the machinery of gut-brain axis, and PAW might be a potential therapeutic strategy in AD.

## INTRODUCTION

Alzheimer’s disease (AD) is the most common form of dementia. AD typically presents with initial symptoms of memory impairment followed by other cognitive dysfunctions, including visuospatial, executive problems and language disturbances [1]. The amyloid-β (Aβ) peptide and hyperphosphorylated tau (p-tau) protein, which are the main components of senile plaques and neurofibrillary tangles respectively, are two pathological biomarkers of AD [2, 3]. Further degeneration in the nucleus basalis of Meynert in cholinergic neuron-rich regions, frontal cortex, anterior and posterior cingulate cortices is associated with memory disturbances, agitation, and apathy [4, 5]. Amyloid positron emission tomography (amyloid-PET) and other biomarker studies implicates that amyloid deposition is the first event of pathological changes in AD and it achieves a significant amount before significant brain atrophy and cognitive symptoms appear [6]. Amyloid precursor protein (APP), Presenilin-1 (PSEN1), presenilin-2 (PSEN2), which are reported to be mutated in familial AD in an autosomal-dominant heredity manner, encode proteins involved in Aβ metabolism [7]. Since the APP gene is located on chromosome 21, trisomy 21 people confers a very high risk of AD, which might be due to the overproduction of Aβ by one more copy of the APP gene [8]. The amyloid hypothesis has been recognized as the key pathogenic mechanism of AD for decades but reducing the amyloid burden in clinical trials failed until 2021. The Aβ-targeted antibody, aducanumab was approved by the US FDA for mild AD. Tau or p-tau protein accumulation, which might be induced by neuronal damage from Aβ, increases along with the clinical progression of AD [9]. Spatial patterns of tau detected by tau PET assays is also closely linked to neurodegeneration and clinical symptoms in AD patients [10]. Recently, amyloid, tau, and neurodegeneration (ATN) have been used as the biomarkers for the precise diagnosis of AD [11].

Patients with AD also exhibit sustained inflammation in the brain. Aβ are suggested to be a proinflammatory agent that activates inflammatory cascades and then damages neurons [12, 13]. Neuroprotective therapies that suppress inflammation, scavenge free radicals or regeneration might prevent neuronal death or brain atrophy induced by inflammation [14]. More importantly, inflammation, on the other hand, also induces Aβ formation and aggregation [15–17].

The gut-brain axes involve bidirectional communication between the enteric and central nervous systems, connecting the cognitive and emotional centers of the brain to the peripheral intestinal functions [18]. The axes are largely influenced by the interactions between the gut microbiota (GM) and both local and systemic immune reactions [19]. Growing evidence supports the role of the GM in activating central microglia and peripheral immune cells [20]. Recent studies have also observed abnormal GM (dysbiosis) in AD and Parkinson’s disease (PD) patients [21]. Dysbiosis was further found to play important roles in blood–brain barrier disruption and Aβ accumulation [22]. Fecal microbiota transplantation (FMT) therapy has been applied to many human diseases, including *Clostridium difficile* colitis, irritable bowel syndrome, PD, autism, and stroke [23–26]. Various GM manipulations, such as germ-free or AD animal-derived GM for wild-type or AD animals, have supported that GM could potentially be a therapeutic target of AD [27]. However, there is still a lack of direct evidence to support the effectiveness of FMT in AD.

On the other hand, engineered water prepared from electro-spraying water vapor is possesses reactive oxygen species (ROS), which can inactivate foodborne microorganisms and can be served as clean alternatives to conventional disinfection methods in food industry [28]. Recently, Zare’s group developed high-speed fused aerosol microdroplets for nanoparticle formation [29]. The micrometer-sized water droplets can induce spontaneous reduction of organic molecules. These innovative aspects suggest that bulk water is inert but treated water without additives can serve as an active solvent. Ohta and his colleagues reported that hydrogen showed the antioxidant activity to reduce oxidative stress in the brain via focal ischemia and reperfusion [30]. Kim and his colleagues reported that electromagnetized gold nanoparticles (AuNPs) can induce dopamine neurons reprogramming for *in vivo* therapy in Parkinson’s disease [31]. Using resonantly illuminated AuNPs, we created stable plasmon-activated water (PAW), which exhibited significantly different properties from the untreated bulk water. PAW shows remarkable antioxidative and anti-inflammatory abilities [32–34]. In our prior report, drinking PAW reduced the amyloid burden, neuroinflammation and improved memory in APPswe/PS1dE9 transgenic mice (AD mice) [35]. These encouraging experimental results indicated the potential application for PAW in neurological diseases. Nevertheless, the mechanisms of the beneficial of PAW on AD are still not totally clear. In this study, the effects of PAW on the GM of AD mice were analyzed. Stool from PAW-feeding AD mice were used for FMT to treat AD mice without PAW. The GM composition, metabolic pathways involved in altered GM, memory behaviors, and pathogenesis-related markers in the intestine and brain of AD mice were analyzed.

## MATERIALS AND METHODS

### Preparation of plasmon-activated water (PAW)

Plasmon-activated water (PAW) was prepared following our previous report [32]. Briefly, sterilized deionized water (DIW) flowed through (300 mL/h) a 30 cm length glass tube filled with sterilized 3 mm ceramic particle-supported gold nanoparticles (AuNPs). Green light-emitting diodes (LEDs, 530 nm) were employed as resonant irradiation to produce PAW.

### Animals and fecal microbiota transplantation (FMT)

APPswe/PSEN1dE9 transgenic mice (AD mice) were purchased from the National Laboratory Animal Center, Taiwan (RRID: MMRRC_034832-JAX; Taipei, Taiwan). The 2–3 mm tail from transgenic mice were used to extract genomic DNA and analyze the genotype following protocol from the Jackson Laboratory using polymerase chain reaction (PCR). Primer sequences were shown as following: oIMR3610 (APP Transgene Forward): 5′-AGGACTGACCACTCGACCAG-3′; oIMR3611 (APP Transgene Reverse): 5′-CGGGGGTCTAGTTCTGCAT-3′; oIMR7338 (IL-2, Internal Positive Control Forward): 5′-CAAATGTTGCTTGTCTGGTG-3′; oIMR7339 (IL-2, Internal Positive Control Reverse): 5′-GTCAGTCGAGTGCACAGTTT-3′. Five-month-old male AD mice and their littermates as controls (wild type) were used in this study. There were 6 groups: wild type with regular water, wild type with PAW, wild type with regular water and FMT, AD with regular water, AD with PAW, AD with regular water, and FMT. Each group contained 6 mice to fed with PAW and water for 10 months at the age of 5 months and changed daily. After one month, stools were continued to collect from PAW-fed and FMT WT and AD mice every day for 10 months. Stool was collected from each group were combined and then separated per 100 mg with normal saline (0.9% NaCl). All the procedures were done in the ice box. After homogenization and centrifugation at 3600 rpm at 4°C for 10 min to collect supernatant. Two hundred microliters of supernatant from every mouse were given to AD or wild-type mice by gavage needles.

### Mouse stool DNA extraction and library construction and sequencing

The QIAamp Fast DNA Stool Mini Kit (Catalog number: 51604; Qiagen, MD, USA) was used to extract tool DNA. 16S ribosomal (rRNA) gene amplification and library construction were performed according to protocols provided by Illumina. The primers 341F: 5′-CCTACGGGNGGCWGCAG-3′; 805R: 5′-GACTACHVGGGTATCTAATCC-3′) was used to amplify the bacterial 16S rRNA genes using polymerase chain reaction (PCR) and sequenced with an Illumina MiSeq sequencer.

### 16S rRNA sequence data analysis and statistical analysis

The universal primers were removed from the demultiplexed paired reads using Cutadapt (v1.12). Sequences were then processed using the package DADA2 (v1.6) in the R environment. The sequences were filtering, trimming and dereplication were independently performed on the forward and reverse reads, and then reads were subjected with the denoising algorithm. And, the paired reads were merged, and remove the chimeras. The inferred ribosomal sequence variants (SVs) were subjected to taxonomic assignment using the SILVA database (v138; http://www.arb-silva.de) as the reference. The multiple sequence alignment of the SVs was performed using by package DECIPHER (v2.6.0), and constructed a phylogenetic tree was by using package phangorn (v2.3.1). A phyloseq object was created, that including the frequency table, taxonomy assignment and phylogenetic tree information. The community analyses were performed using package phyloseq (v1.19.1). Mann–Whitney *U*-test was performed to detect differentially abundant taxonomic ranks between the difference groups. The sample relatedness was analyzed by UniFrac distances calculated using the GUniFrac package (v1.1) to assess the community dissimilarity between difference groups [36]. Principal coordinate analysis ordination of UniFrac distances was performed, and the adonis and betadisper functions were analyzed by the vegan package (v2.4) for statistically analyze the dissimilarity of compositions among groups and the homogeneity of dispersion. Moreover, the microbiota enrichment analysis between groups was analyzed by the method of linear discriminant analysis effect size (LEfSe) and with alpha set to 0.05 (Kruskal–Wallis and Wilcoxon tests) and the LDA score of 2 or more [37] and visualized by using package GraPhlAn [38].

### The prediction of functional changes associated with microbiota

The potential functional changes by the microbiota were predicted by using Phylogenetic Investigation of Communities by Reconstruction of Unobserved States (PICRUSt2) [39]. The relative predicted abundance of MetaCyc pathways was calculated by dividing the abundance of each pathway by the sum of all pathway abundances per sample [40]. The relative contribution of each operational taxonomic unit (OTU) to the predicted pathways was calculated by dividing the contribution of each OTU by the sum of all contributions per sample.

### Novel object recognition (NOR) test

The NOR test followed the procedures of our earlier report [35]. Briefly, 16-month-old mice were analyzed by the NOR test in a black box using a camera to monitor the behavior of the mice, which were trained and habituated. Each mouse was explored the environment for 15 min in the box from days 1 to day 3. Each mouse was placed in the box containing two identical objects for 15 min and then return to its cage on day 3. The mouse was then placed back into the box containing one novel object and one familiar object. Using camera to record the mouse for 15 min. The video was used to trace the mouse by Noldus EthoVision XT (Noldus, VA, USA). An NOR index was used to evaluate. NOR % = ((seconds of novel object)/(seconds of novel object + seconds of familiar object)). Each data was obtained from three independent experiments and analyzed by the mean ± standard deviation (SD).

### Morris water maze (MWM) test

The MWM test was conducted by following the procedures of a previous report [41]. Briefly, mice were trained in a circular pool (120 cm in diameter and 50 cm in deep) filled with water (at 20~22°C). The pool was divided into four equal quadrants (the right upper, right lower, left upper and left lower) that had different marks on the wall. A platform was placed 1.5 cm below the surface of the water in the left upper quadrant. For the training process, a mouse was placed into the water facing the wall in the left upper, left lower, right lower, and right upper quadrants and trained six times each day for 3 days. The mouse was allowed to swim freely until it reached the hidden platform. If a mouse failed to find the platform after 60 s, it was placed on the platform for 20 s. After training for 3 days, the mouse was placed in the water in the right lower quadrant, and the length of time to find the platform was recorded (latent time).

### ^18^F-florbetapir positron emission tomography (PET) imaging

The procedures of the PET assay followed our earlier report [35]. Briefly, ^18^F-florbetapir (AV-45/Amyvid) was synthesized for amyloid PET analysis. Animal PET was conducted in 16-month-old AD and C57BL/6 mice (WT; *n* = 6 per treatment group) using a preclinical Inveon PET system (Siemens Medical Solutions, Knoxville, TN, USA). After injecting ^18^F-florbetapir (19.52 ± 1.06 MBq in 0.1 mL of saline) through a tail vein, each mouse was then subjected to isoflurane anesthesia (1.5% in oxygen gas) for a 60-min dynamic scan. All images were analyzed using PMOD image analytical software (vers. 3.7, PMOD Technologies Ltd, Zurich, Switzerland). Each mouse PET image with the PMOD built-in T1-weighted magnetic resonance imaging (MRI) template was co-registered to ensure proper placement of the volume of interest (VOI). The built-in volumes of interest (VOIs) of the cortex, striatum, and hippocampus from the MRI template were fused with PET images for quantification. After correction for radioactive decay, mean ^18^F-florbetapir activities were evaluated for each VOI on integrated PET images recorded over 30~60 min. Standardized uptake values (SUVs) of ^18^F-florbetapir in each VOI were calculated by dividing the mean radioactivity counts by the injected dose and body weight. SUV ratios (SUVRs) were calculated as SUVcortical/SUVcerebellum, and the cerebellum was used as the reference region.

### Immunofluorescent staining

Mice were anesthetized with 2% isoflurane (Attane, Panion and BF Biotech Inc., Taipei, Taiwan) and sacrificed by cardiac perfusion with 4% paraformaldehyde (Catalog number: P6148; Sigma). Brains were collected and incubated for post-fixation at 4°C overnight. Brain tissue samples were cut into 5-μm sections to make parafilm slices. Slices were deparaffinized and dehydrated by incubation with Nova Histo (Catalog number: LB0200-0200; Bionovas, Toronto, Canada) in a microwave machine for 3 min. After washing with deionized water several times, brain slides were then blocked with 5% bovine serum albumin (BSA) in PBST (0.1% Tween-20 in phosphate-buffered saline (PBS). Slices were incubated with monoclonal antibodies against p-tau (Catalog number: MN1020; AT8, Thermo Fisher, MA, USA), IL-1β (Catalog number: 12242; Cell Signaling, MA, USA), IL-6 (Catalog number: 12912; Cell Signaling), TNF-α (Catalog number: 11498; Cell Signaling), and βA (Catalog number: MAB1501; Millipore, CA, USA) at 4°C overnight. After washing with PBST three times, slides were incubated with an Alexa 488-conjugated secondary antibody for 1 h. Images were analyzed by TissuFAXS (Tissuegnostics, Vienna, Austria).

### Western blot analysis

Brain tissue samples were dissected into cortex and hippocampus before frozen in liquid nitrogen, and then stored at −80°C until use. The frozen cortical and hippocampal tissue samples were homogenized in 1 mL of tapered tissue grinders in 5-fold of ice-cold RIPA buffer (Catlog number: 20-188; Millipore) with a protease inhibitor cocktail (100X, Catalog number: P8340; Sigma) and a phosphatase inhibitor (100X, Catalog number: PIC008.1; BioShop, Canada). After quantifying the protein content using the Bradford protein assay (Catalog number: 5000205; Bio-Rad, CA, USA), 20 μg of protein was separated using an 4-20 % sodium dodecylsulfate (SDS) polyacrylamide gradient gel (Catalog number: TFU-GG420; TOOLS, Taiwan), and then transferred to a polyvinylidene difluoride (PVDF) membrane (Catalog number: BSP0861; Pall, FL, USA). The membrane was blocked with BLOCKPRO blocking buffer (Catalog number: BM01-500; VISUAL PROTEIN, Taiwan) for 30 min, followed by incubation with a primary antibody at 4°C overnight. The antibody concentration of IL-1β (Catalog number: 12242; Cell Signaling, MA, USA), IL-6 (Catalog number: 12912; Cell Signaling), and TNF-α (Catalog number: 11498; Cell Signaling) used for western blotting were 2, 4, and 0.8 μg/mL. After washing with PBST three times, membranes were incubated with horseradish peroxidase (HRP)-conjugated secondary antibodies. Anti-p-tau (Catalog number: GTX50171) and anti-tau (Catalog number: GTX50451) antibodies were purchased from GeneTex (GeneTex, Taiwan). Anti-β-amyloid and amyloid precursor protein antibody (Catalog number: SIG-39300) were purchased from BioLegend (BioLegend, CA, USA). An anti-beta-actin antibody was purchased from Millipore (Catalog number: MAB1501). Anti-arginase 1 (Catalog number: ab259271) and anti-CD86 (Catalog number: ab220188) antibodies were purchased from Abcam. Goat anti-mouse immunoglobulin G (IgG)-HRP (Catalog number: sc-2031) and anti-rabbit IgG-HRP (Catalog number: sc-2357) antibodies were purchased from Santa Cruz Biotechnology (Santa Cruz, CA, USA). Protein signals were analyzed using a Western Lightning Enhanced Chemiluminescence Substrate kit (Catalog number: NEL103E001EA; Perkin Elmer, MA, USA) and BioSpectrum AC Imaging System (UVP, CA, USA). Protein levels were quantified with UVP Vision Works software.

### Enzyme-linked immunosorbent assay (ELISA) for IL-1β, IL-6, and TNF-α

Total proteins were extracted from frozen cortical and hippocampal tissue samples by homogenization in tapered tissue grinders with cold RIPA buffer (Millipore) containing protease (Sigma) phosphatase inhibitor (BioShop). The Bradford protein assay (Catalog number: 500-0006; Bio–Rad) was used to measure the protein concentration. Quantification of IL-1β, IL-6, and TNF-α was performed using cytokine detection kits (DY401, DY406, and DY410, respectively, R&D Systems, MN, USA) according to the manufacturer’s protocol. All samples were used 250 μg/mL for ELISA. The detection range was indicated as IL-1β (15.6–1,000 pg/mL), IL-6 (15.6–1,000 pg/mL), and TNF-α (31.2–2,000 pg/mL). A SpectraMax microplate reader was used to measure absorbance at 450 nm (Molecular Devices, CA, USA). Duplicate replicates were analyzed for samples.

### Immunohistochemistry

Brain and jejunal tissues were collected from mice and then perfused with 4% paraformaldehyde. The 5-um parafilm slides were cut from tissues and deparaffinized with Trilogy (Catalog number: 920P-04; Cell Marque Corporation, AR, USA) at 120°C for 15 min and washed with tap water several times. Slides were blocked with PBS containing 0.5% Triton X-100 (Catalog number: 11332481001, Sigma) and 5% BSA. Slides were incubated with primary antibody, including anti-Occludin (Catalog number: ab216327), anti-Claudin-5 (Catalog number: ab131259), and anti-Axl (Catalog number: ab219651) antibodies (Abcam) antibodies overnight. Secondary antibody (Catalog number: K4065; Agilent, CA, USA) conjugated with a peroxidase-labeled polymer was incubated for 30 min and then stained with a Dako REAL EnVision Detection system (Catalog number: K500711-2; Agilent) for 5 min. Capture images were analyzed by MoticEasyScan Pro 6 Imaging System (Motic, TX, USA).

### Statistical analysis

All data were collected from three independent experiments and analyzed by mean ± standard deviation (SD). A one-way analysis of variation (AVOVA) followed by Tukey’s test was used to analyze the statistical significance using R software (version 4.0.3) and indicated by ^*^p < 0.05, ^**^p < 0.01, and ^***^p < 0.001.

### Data availability

The data supporting the conclusions is all contained within the manuscript and supplementary information.

## RESULTS

### Gut microbiota profiles were different between regular water-fed AD and PAW-fed AD mice

**Figure 1:**
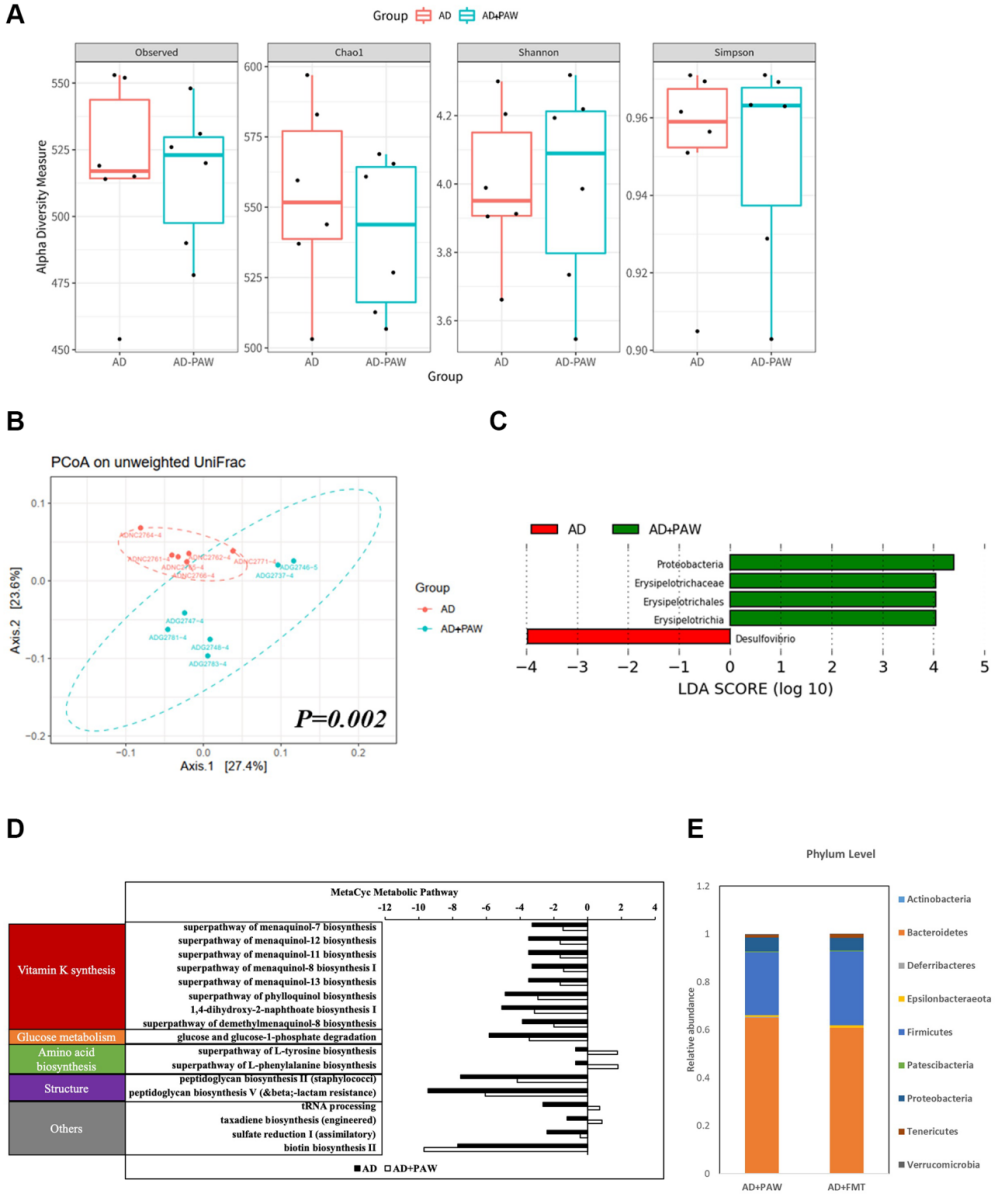
**Fecal microbiome distribution analysis in AD- and plasmon-activated water (PAW)-fed AD mice.** The stool from AD and PAW-fed AD mice were prepared for fecal microbiotic profiling by high-throughput sequencing of the 16S rRNA gene with the Illumina MiSeq system. (**A**) Alpha diversity of PAW-treated samples and untreated controls. Principal coordinate analysis (PCoA) plot based on (**B**) unweighted UniFrac distances of PAW-treated samples and untreated controls. Significant differences in beta diversity were evaluated with a permutational multivariate analysis of variance (vegan::adonis, 1000 permutations), and beta dispersion was quantified with a betadisper (vegan::betadisper, 1000 permutations). The PCoA unweighted UniFrac indices achieved adonis *p* < 0.05 and betadisper *p* > 0.05. (**C**) Linear discriminant analysis (LDA) effect size (LEfSe) analysis of gut microbiotic changes in mice treated with PAW. Significant biomarkers were defined as taxa with an LDA score (log10) of  ≥ 2. (**D**) Prediction of PAW-fed induced metagenome functions change in AD mice based on 16S sequencing analysis by PICRUSt2 analysis. (**E**) Comparison of fecal microbiotic distributions in PAW-fed and fecal microbiota transplantation (FMT) AD mice.

The gut microbiota (GM) of wild-type (WT) and APPswe/PS1dE9 mice (AD mice) was differed from each other (Supplementary Figure 1). The alpha diversity of the GM of PAW-fed AD mice (AD+PAW mice) were not significantly different from those of regular water-fed AD mice (AD mice) (Figure 1A). This result indicates that PAW does not cause significant changes in the enrichment of gut microbiota. In beta diversity, Unweighted UniFrac principal component analysis (PCoA) showed that AD+PAW mice displayed a different fecal microbiome profile (*p*-value = 0.002) than regular AD mice (Figure 1B). This result suggests that PAW causes changes in gut microbiota composition and there was a statistically significant difference between the two groups. The results of a taxonomic analysis of the microbiota showed that the genera *Desulfovibrio* decreased and the family *Erysipelotrichaceae*, phylum Proteobacteria increased in AD+PAW mice (Figure 1C). Similar profiles also observed in comparison of WT with AD mice (Supplementary Table 1). In addition, we further predicted the functional capacity of the gut microbiota by using inference based on 16S rRNA gene amplicon sequences (PICRUSt2). The relative abundance of all predicted pathways suggested major differences in the functional potential of the microbiota between AD and AD+PAW mice. The functions were grouped into vitamin K synthesis, glucose metabolism, amino acid biosynthesis, structure, and others. Most abundant microbiota in these groups was vitamin k synthesis (Figure 1D). Furthermore, to further confirm the microbiota profile was altered by PAW, we collected stools from AD+PAW mice for fecal microbiota transplantation (AD+FMT mice). We collected subjected those samples to 16S rRNA next-generation sequencing (NGS). The results showed that bacteria taxonomy had high similarities and abundances between AD+PAW and AD+FMT mice (Figure 1E). PAW treatment also produced a similar profile, including *Desulfovibrionaceae_ge, Desulfovibrio, Erysipelatoclostridium, Candidatus_Stoquefichus, Allobaculum, Ruminococcus_1, Akkermansia, Mucispirillum, Lachnospiraceae_FCS020_group, Tyzzerella,* and *Helicobacter,* in WT mice compared to the AD model (Supplementary Table 2). These results suggested that AD+FMT mice could stably colonize the gut microbiota from AD+PAW mice.

### Memory declines were ameliorated in FMT- and PAW-fed AD mice

**Figure 2:**
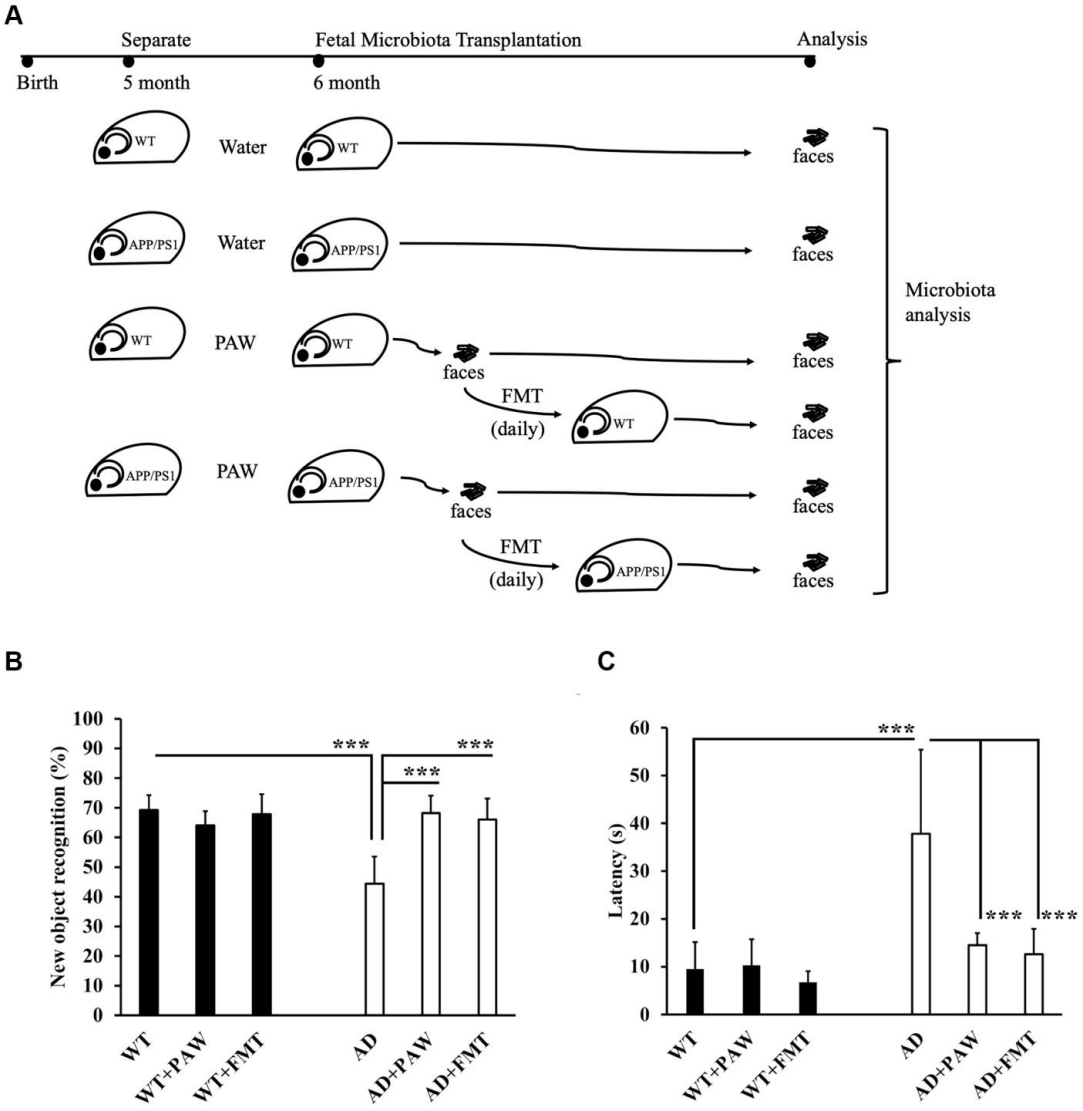
**Memory function assessment of plasmon-activated water (PAW)-fed and fecal microbiota transplantation (FMT) AD mice.** (**A**) The schema shows the timeline of wild-type (WT) and AD mice fed daily water, PAW and FMT of stool from 6-month-old PAW-fed AD mice. Memory function assessment of 16-month-old WT, WT-fed PAW (WT+PAW), FMT (WT+FMT), AD, PAW-fed AD (AD+PAW), and FMT AD (AD+FMT) mice by a novel object recognition test (**B**) and Morris water maze (**C**) test. All results were analyzed by ANOVA with a post hoc analysis. (^*^p < 0.05, ^**^p <0.01, ^***^p <0.001). Mean values ± standard deviation of mean (SD) was shown.

To test the effects of PAW and FMT from PAW AD mice on the amelioration of memory decline, we prepared AD and wildtype (WT) mice to be fed PAW (WT+PAW, AD+PAW), FMT (WT+FMT, AD+FMT), and regular water (WT, AD) from 5 to 16 months of age (Figure 2A). The NOR index of AD mice decreased from 69% to 44% compared to WT mice according to an NOR assay. The AD+PAW and AD+FMT mice exhibited a resurgence in the recognition index from 44% to 68% and 66%, respectively, which were levels like those of WT mice. No significant differences were observed in WT, WT+PAW, or WT+FMT mice (Figure 2B). In the Morris water maze assay, AD mice showed significantly prolonged latency at 38 s compared to 10 s for WT mice. The AD+PAW mice showed significant reductions in latency to 15 and 13 s, respectively, which were similar to values for WT mice. No significant differences were observed among WT, WT+PAW, or WT+FMT mice (Figure 2C). These results showed that PAW and FMT might reverse the AD symptoms in AD mice.

### Radioactivity uptake was reduced in FMT- and PAW-fed AD mice

**Figure 3:**
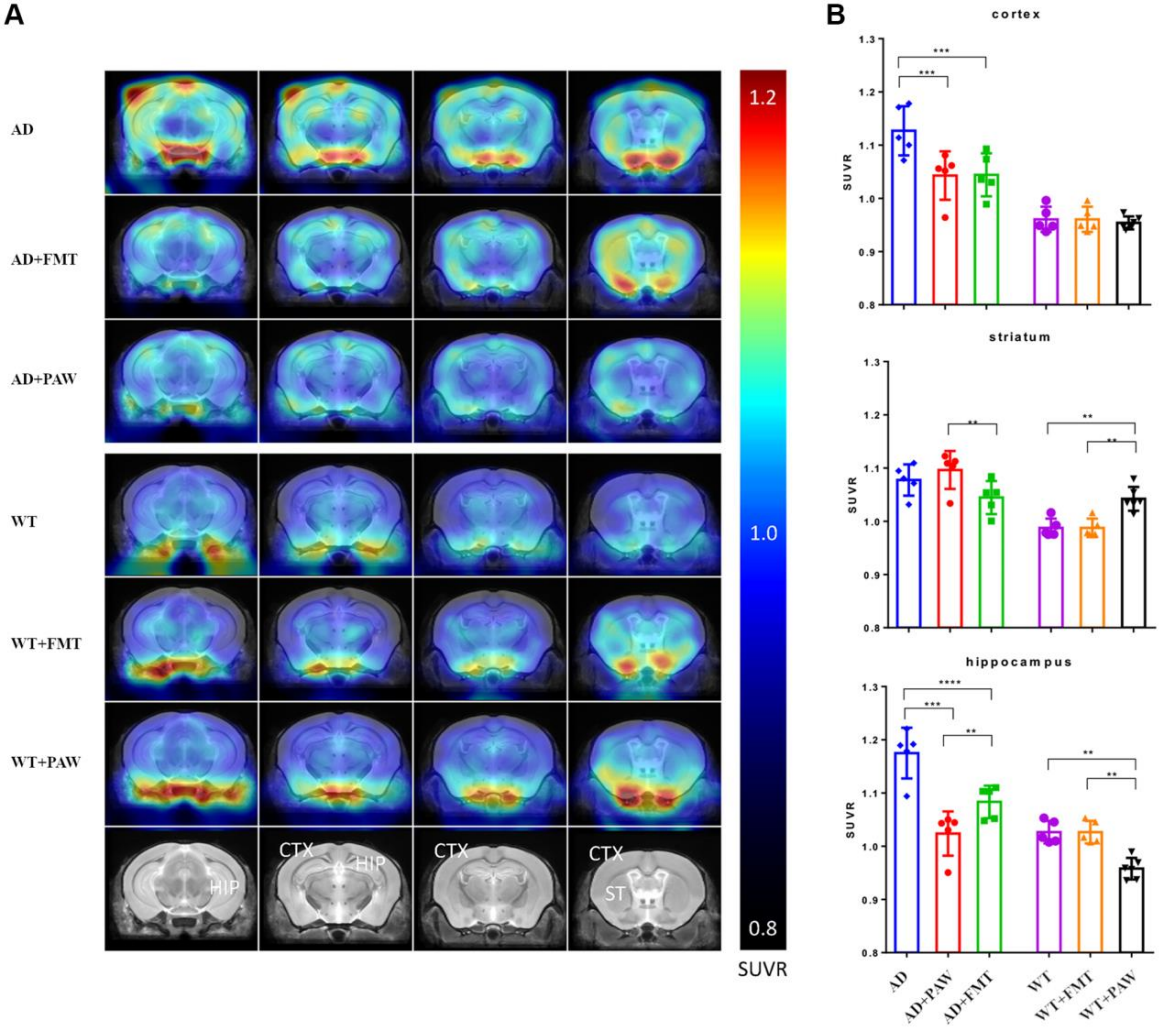
**Radioactivity uptake assay of fecal microbiota transplantation (FMT) and plasmon-activated water (PAW)-fed AD mice.** (**A**) 18F-Florbetapir animal positron emission tomographic (PET) images were generated from 16-month-old wild-type (WT), WT fed PAW and FMT WT, AD, PAW-fed AD, and FMT AD mice. The color bar of the right line shows the standardized uptake value ratio (SUVR) compared to the cerebellum (SUVRcer). (**B**) Comparison of relative 18F-florbetapir uptake levels in the cortex, striatum, and hippocampus of the brain. All results were analyzed by ANOVA with a post hoc analysis. (^*^p < 0.05, ^**^p < 0.01, ^***^p < 0.001). Mean values ± standard deviation of mean (SD) was shown.

To explore the mechanisms of the rescue of memory declines in AD+FMT mice, we conducted amyloid PET to measure changes in amyloid accumulation in the brains of wildtype (WT) and AD mice fed with water (WT, AD), PAW (WT+PAW, AD+PAW) and FMT (WT+FMT, AD+FMT). Intense and diffusely increased uptake of radioactivity was observed in the cortex and hippocampus of AD mice. The AD+PAW and AD+FMT mice exhibited significantly reduced uptake of radioactivity in the cortex and hippocampus compared to AD mice (Figure 3A, 3B). The standardized uptake value (SUV) ratios (SUVRs) in the cerebellum (SUVRcer) of each brain region are shown in Supplementary Table 3. These results suggest that PAW and FMT experienced a reduction of amyloid accumulation in the brain of AD mice.

### Aβ and p-tau burdens decreased in the cortex and hippocampus of FMT- and PAW-fed AD mice

**Figure 4:**
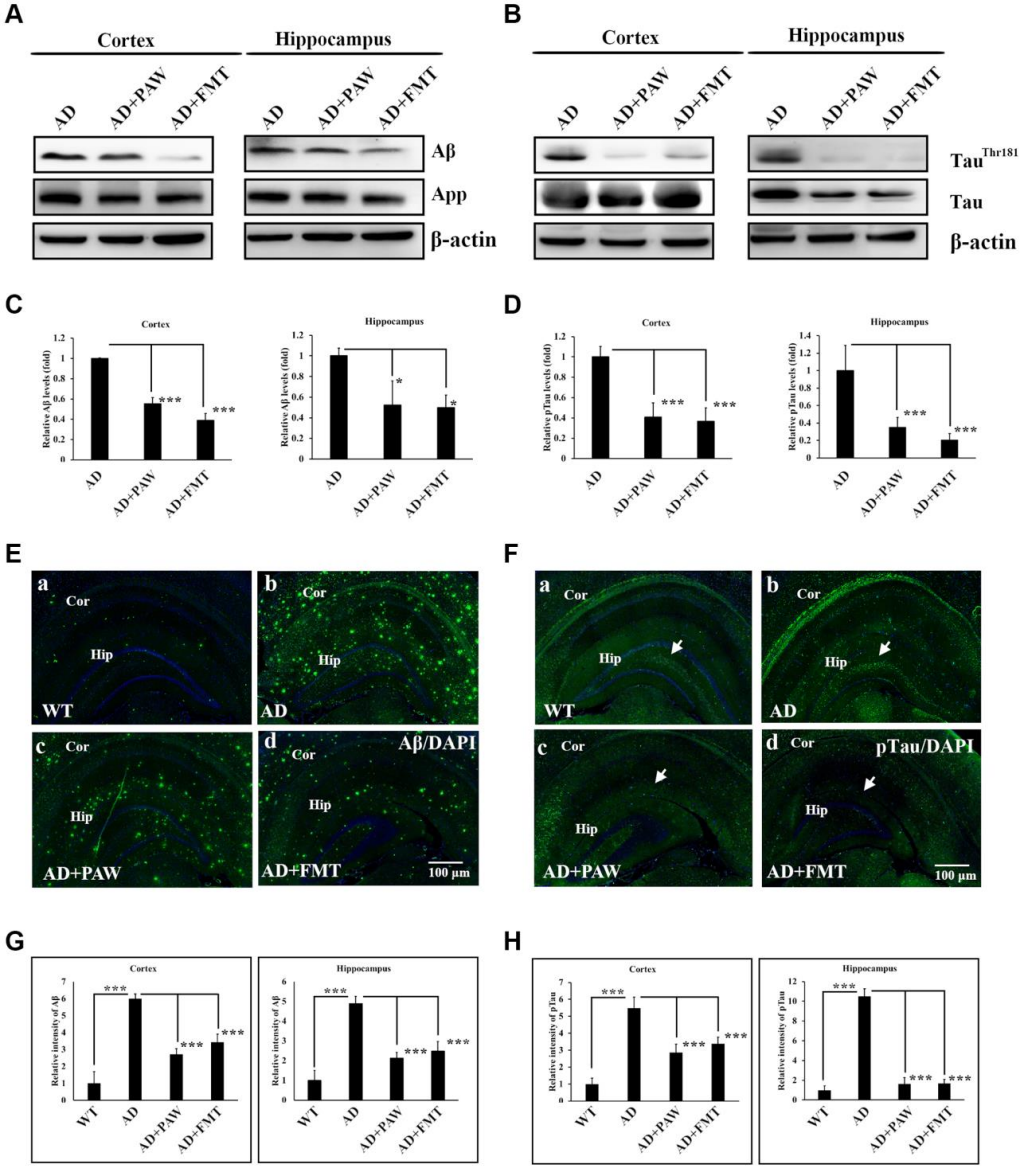
**Amyloid and phosphorylated (p)-tau burden analysis in the cortex and hippocampus of AD mice.** Protein levels of amyloid precursor protein (APP), amyloid beta (Aβ) (**A**), p-tau (Tau^Thr181^), and tau protein (**B**) in the cortex and hippocampus of AD mice, plasmin-activated water (PAW)-fed (AD+PAW) mice, and fecal microbiota transplantation (FMT) AD mice (AD+FMT) as measured by Western blotting. Statistical results (**C**) and (**D**) were analyzed from (**A**) and (**B**). Immunofluorescence analysis of Aβ (**E**) aggregation in the cortex (Cor) and hippocampus (Hip) of WT (**a**), AD (**b**), AD+PAW (**c**), and AD+FMT mice (**d**). Immunofluorescence analysis of p-tau (**F**) aggregation in the cortex (Cor) and hippocampus (Hip) of WT (**a**), AD (**b**), AD+PAW (**c**), and AD+FMT mice (**d**). Statistical results (**G**) and (**H**) were analyzed in the cortex and hippocampus from (**E**) and (**F**). All results were analyzed by ANOVA with a post hoc analysis. (^*^p < 0.05, ^**^p < 0.01, ^***^p < 0.001). Mean values ± standard deviation of mean (SD) was shown.

Aβ and p-tau burdens are the most important biomarkers of AD [42]. In our earlier report, we found reductions in Aβ and p-tau in the brains of PAW-fed mice [35]. To further analyze the Aβ and p-tau burdens in PAW (AD+PAW) and FMT (AD+FMT) mice, we examined the protein levels of Aβ and p-tau by Western blotting analysis and immunofluorescence (IF) staining. Protein levels of Aβ significantly decreased in the cortex and hippocampus of both AD+PAW and AD+FMT mice compared to regular water-fed AD mice (Figure 4A, 4C). Levels of p-tau were also reduced in the cortex and hippocampus of AD+FMT and AD+PAW mice compared to AD mice (Figure 4B, 4D). Immunofluorescent (IF) staining also showed the reduction of Aβ (Figure 4E, 4G) and p-tau (Figure 4F, 4H) aggregation in the cortex and hippocampus. These results suggest that FMT mice showed similar effects of reducing the amyloid and p-tau burdens in the brains of PAW-fed AD mice.

### Inflammation might be reduced in the hippocampus of FMT- and PAW-fed AD mice

**Figure 5:**
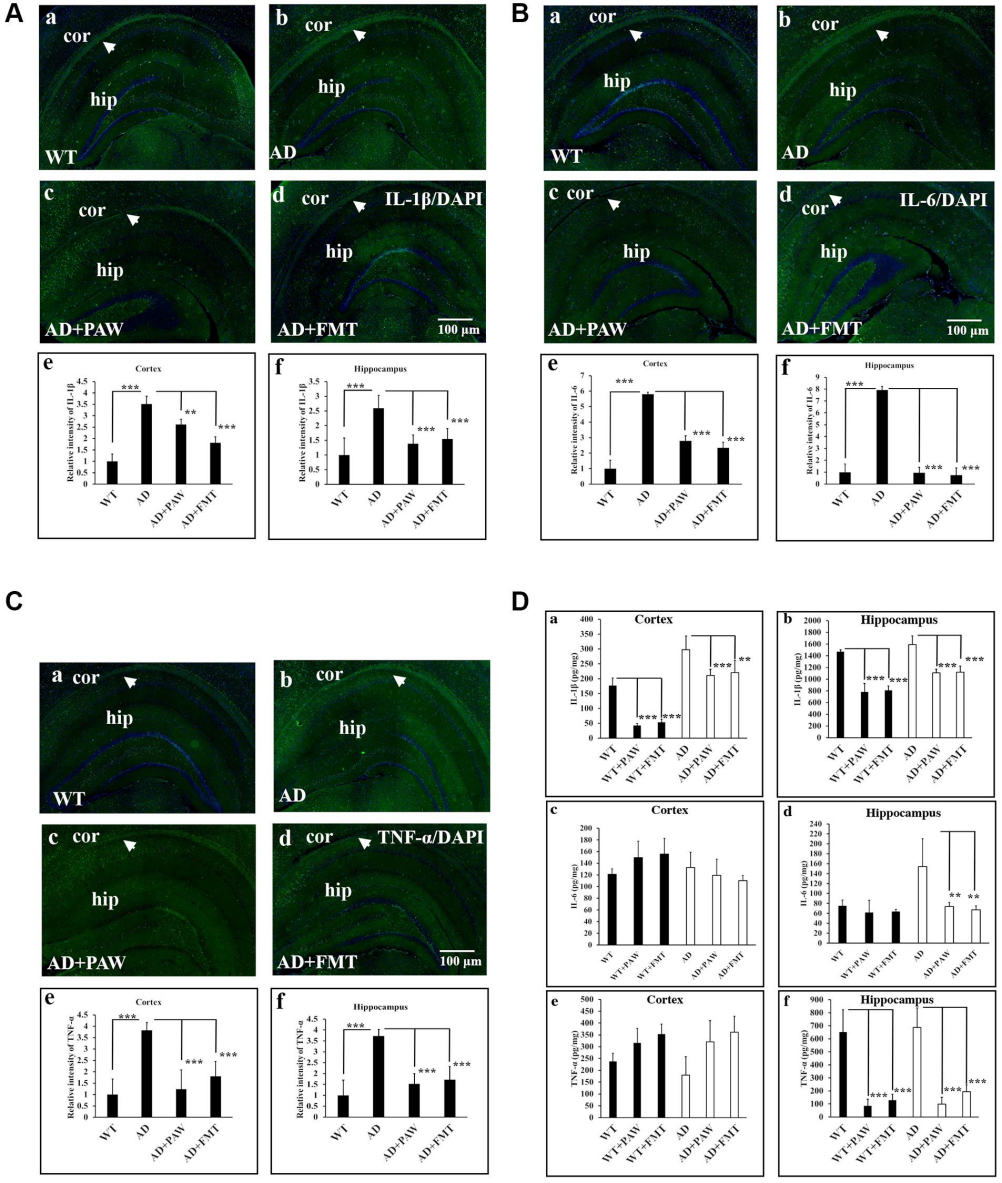
**Inflammation marker analysis in the cortex (Cor) and hippocampus (Hip) of wild-type (WT) and AD mice.** Immunofluorescence analysis of interleukin (IL)-1β (**A**), IL-6 (**B**), and tumor necrosis factor (TNF)-α (**C**) in the Cor and Hip of WT mice (**a**), AD mice (**b**), plasmin-activated water (PAW)-fed (AD+PAW) mice (**c**), and fecal microbiota transplantation (FMT) AD mice (AD+FMT) (**d**). Statistical results were analyzed in the Cor (**e**) and Hip (**f**) from (**A**), (**B**), and (**C**). (**D**) Protein concentrations of IL-1β (**a**, **b**), IL-6 (**c**, **d**), and TNF-α (**e**, **f**) from the Cor and Hip were measured by ELISA of WT, WT+PAW, WT+FMT, AD, AD+PAW, and AD+FMT mice. All results were analyzed by ANOVA with a post hoc analysis. (^*^p < 0.05, ^**^p < 0.01, ^***^p < 0.001). Mean values ± standard deviation of mean (SD) was shown.

Inflammation is generally detected in the pathologically vulnerable regions of AD patients. We analyzed the protein levels of inflammatory markers in the cortex and hippocampus of AD mice by IF staining and an enzyme-linked immunosorbent assay (ELISA). The protein levels of IL-1β (Figure 5A), IL-6 (Figure 5B), and TNF-α (Figure 5C) were significantly increased in AD mice (Figure 5Ab, 5Bb, 5Cb, 5Ae, 5Af, 5Be, 5Bf, 5Ce, 5Cf) and decreased in PAW-fed (AD+PAW) (Figure 5Ac, 5Bc, 5Cc, 5Ae, 5Af, 5Be, 5Bf, 5Ce, 5Cf) and FMT (AD+FMT) (Figure 5Ad, 5Bd, 5Cd, 5Ae, 5Af, 5Be, 5Bf, 5Ce, 5Cf) AD mice compared to water-fed AD mice (Figure 5Aa, 5Ba, 5Ca, 5Ae, 5Af, 5Be, 5Cf, 5Ce, 5Cf), as measured by an IF staining assay (Figure 5A–5C). Further analysis of the protein levels of IL-1β, IL-6, and TNF-α in the hippocampus of AD mice by ELISA revealed a reduction in protein levels in AD+PAW and AD+FMT mice compared to water-fed AD mice (Figure 5Db, 5Dd, 5Df). In the cortex, the IL-1β protein concentration was reduced in AD+PAW and AD+FMT mice (Figure 5Da). The protein levels of IL-6 (Figure 5Dc), and TNF-α (Figure 5De) in the cortex were not changed. Protein levels of inflammatory markers measured by ELISA are shown in Supplementary Table 4. These results suggest that PAW and FMT can possibly reduce inflammation in the hippocampus of AD mice.

### M1/M2 ratio was increased in PAW-fed and FMT AD mice

**Figure 6:**
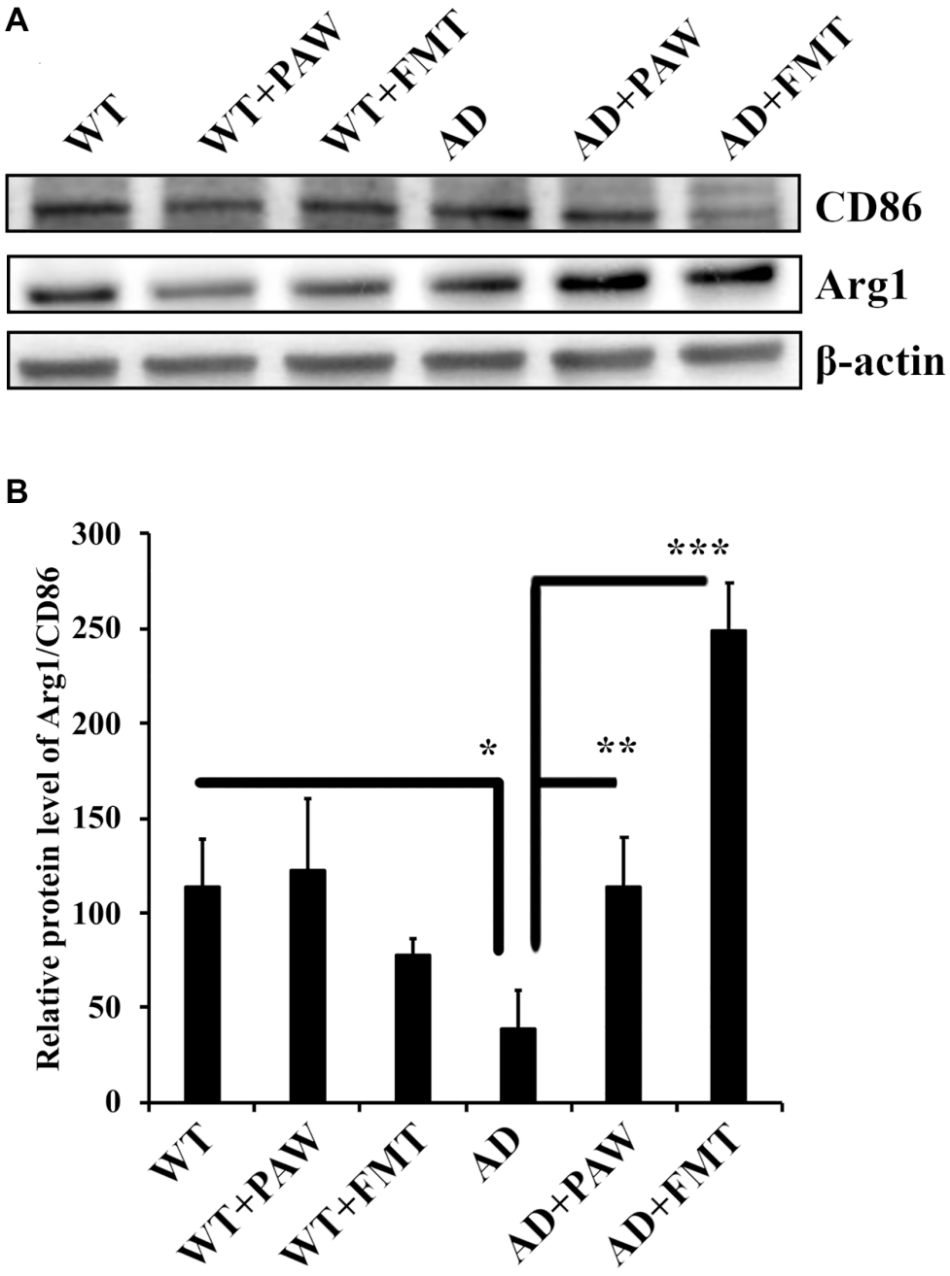
**Protein levels of arginase-1 and CD86 in the cortex of wild-type (WT) and AD mice.** (**A**) Western blot analysis of arginase-1 (Arg1) and CD86 in the cortex of WT and AD mice fed PAW (WT+PAW, AD+PAW) and FMT (WT+FMT and AD+FMT). (**B**) Protein levels of Arg1 and CD86 were quantified from (**A**). All results were analyzed by ANOVA with a post hoc analysis. (^*^p < 0.05, ^**^p < 0.01, ^***^p < 0.001). Mean values ± standard deviation of mean (SD) was shown.

Neuroinflammation induced by hypoxia activates M1 microglia and reduces M2 microglia by determining CD86 and Arginase-1 expression in Alzheimer’s disease [43]. To investigate the effects of PAW and FMT-induced microbiota alterations on neuroinflammation in AD mice, we analyzed the marker protein levels of M1 (CD86) and M2 (Arginase-1) microglia in brain tissue. The M2/M1 ratio was reduced in AD mice, but it was reversed in AD mice fed with PAW (AD+PAW) or subjected to FMT (AD+FMT) AD mice (Figure 6A, 6B). These findings suggest that PAW and FMT have the potential to reverse neuroinflammation in AD mice.

### Epithelial barrier integrity was increased by microbiota alterations in PAW-fed and FMT AD mice

**Figure 7:**
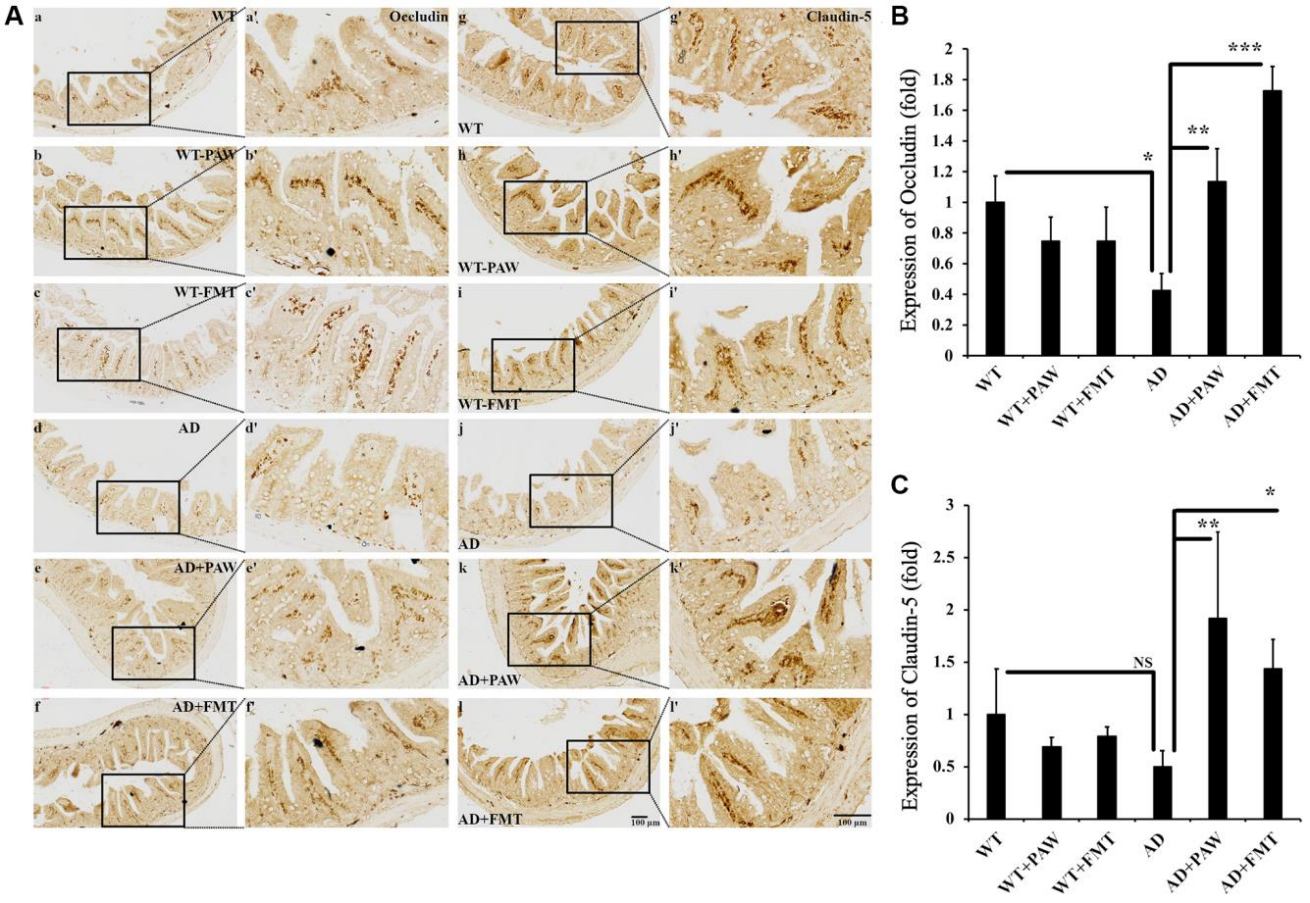
**Immunohistochemistry analysis of the protein levels of Occludin and Claudin-5 in the jejunum of WT and AD mice.** (**A**) The jejunum from WT (**a**–**c**, **g**–**i**) and AD (**d**–**f**, **j**–**l**) mice fed PAW (**b**, **h**, **e**, **k**) and FMT (**c**, **I**, **f**, **l**) was analyzed by immunohistochemistry with anti-Occludin (**a**–**f**) and anti-Claudin-5 (**g**–**l**) antibodies. Images (**a’**–**l’**) were enlarged from images (**a**–**l**). Quantitation of the protein levels of Occlusion (**B**) and Claudin-5 (**C**) from (**A**) was analyzed. All results were analyzed by ANOVA with a post hoc analysis. (^*^p < 0.05, ^**^p < 0.01, ^***^p < 0.001). Mean values ± standard deviation of mean (SD) was shown.

Alzheimer’s disease has been reported to correlate with breakdown of gut epithelial barrier integrity, increased permeability due to dysbiosis-induced changes in gut microbiota, and neuroinflammation-induced symptoms [44]. To analyze the epithelial barrier integrity in PAW-fed and FMT AD mice, we conducted immunohistochemical analysis to measure the levels of occludin and claudin-5 [45, 46], which are known markers of gut epithelial integrity. The levels of occludin protein were found to be decreased in AD mice compared to WT mice (Figure 7A, 7B), but they were reversed in AD mice fed with PAW (AD+PAW) or subjected to FMT (AD+FMT) (Figure 7Aa–f, a’–f’, 7B). The levels of claudin-5 protein were also decreased in AD mice compared to WT mice, but this finding did not reach statistical significance. In AD mice fed with PAW (AD+PAW) or subjected to FMT (AD+FMT), the levels of claudin-5 protein were significantly increased compared with those in AD mice (Figure 7Ag–7l, 7g’–7l’, 7C). These results suggest that PAW and FMT may increase the gut epithelial barrier integrity in AD mice.

### AXL was activated in the brain of PAW and FTM mice

**Figure 8:**
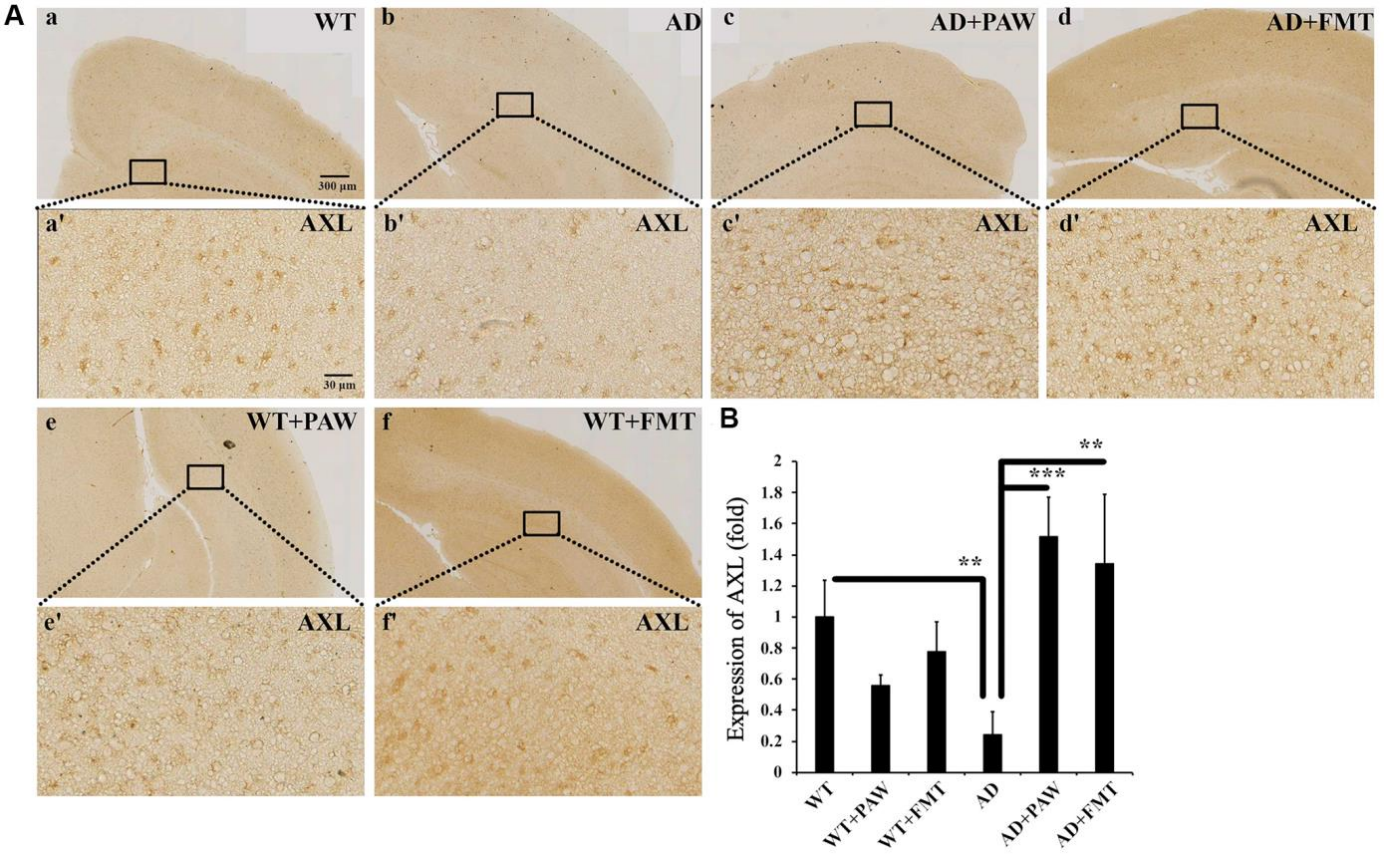
**Immunohistochemistry analysis of the protein levels of AXL in the cortex of WT and AD mice.** (**A**) The brains from WT (**a**, **e**, **f**) and AD (**b**, **c**, **d**) mice fed water (**a**, **b**), PAW (**c**, **e**), and FMT (**d**, **f**) were analyzed by immunohistochemistry with anti-AXL. Images (**a’**–**f’**) were enlarged from images (**a**–**f**). (**B**) Protein levels of AXL were quantified from (**A**). All results were analyzed by ANOVA with a post hoc analysis. (^*^p < 0.05, ^**^p < 0.01, ^***^p < 0.001). Mean values ± standard deviation of mean (SD) was shown.

In earlier reports, vitamin K deficiency was found to contribute to the pathogenesis of Alzheimer’s disease, and supplementation with vitamin K might have a beneficial effect on prevention or treatment [47, 48]. Vitamin K was shown to activate the AXL pathway and reduce neuroinflammation in the brain [49, 50]. In our present study, microbiota profiles were altered by PAW and FMT to group functions on vitamin K synthesis. To further analyze the activation of AXL by PAW and FMT in AD mice, the protein levels of AXL in the brain were measured by immunohistochemistry. The protein levels of AXL were reduced in AD mice (Figure 8Ab, 8b’, 8B) compared to WT mice (Figure 8Aa, 8a’, 8B) but they were reversed in PAW-fed (AD+PAW) (Figure 8Ac, 8c’, 8B) and FMT (AD+FMT) AD mice (Figure 8Ad, 8d’, 8B). There was no significant difference in AXL protein expression between PAW-fed (WT+PAW) mice (Figure 8Ae, 8e’, 8B) and FMT (WT+FMT) mice (Figure 8Af, 8f’, 8B) compared to WT mice (Figure 8Aa, 8a’, 8B). These results suggest that AXL expression might be activated in the brain of PAW-fed and FMT AD mice.

## DISCUSSION

Previously, we found that PAW reduced the brain amyloid burden and improved the cognitive function in AD mice. However, the underlying neuroprotective mechanisms are unclear. In this study, we found that PAW altered GM profiles in AD mice, especially anti-proinflammatory-related bacterial profile, including an increase in *Erysipelotrichaceae* increases and a decrease in *Desulfovibrio* decreases. In addition, FMT treatment of AD mice with stool from PAW-feeding AD mice resulted in almost identical GM profiles to those of PAW-fed AD mice. Both treatments demonstrated improvements in cognitive function and a reduction in brain amyloid burden in AD mice. In addition, both treatments resulted in the preservation of intestinal epithelial integrity, inactivation of proinflammatory microglia, and reduction of proinflammatory cytokines in the brains of AD mice. Additionally, the expression of the vitamin K-dependent anti-inflammatory protein Axl was increased in the brains of PAW-feeding and FMT AD mice. In summary, we provide evidence that PAW improved cognitive functions and reduced the brain amyloid burden through the change in GM composition and then subsequent reduction of neuroinflammation. Therefore, we have also shown that GM should play an important role in pathophysiology of AD and microbiota-targeted interventions by PAW could be a potentially effective strategy to delay AD occurrence and progression.

Many studies have documented that GM compositions are different in AD patients and animals from non-AD individuals [22, 51, 52]. The GM from AD patients or transgenic AD animals showed reduced diversity or richness and more dominance of *Helicobacter* and less dominance of *Bacteroides* compared to non-AD controls or WT animals [53]. Our results are compatible with these prior reports. Additionally, this study showed that PAW altered the composition of the GM microbiome but did not lead to changes in total richness of AD mice. Furthermore, increased the diversity of the GM of AD mice but also increased *Erysipelotrichaceae* and suppressed proinflammatory *Desulfovibrio* microbes. Although the linkage between *Helicobacter* infection and AD is still controversial, seropositivity of *Helicobacter pylori* is associated with AD mortality, all-cause dementia, and AD dementia among men and higher-socioeconomic-status individuals in large national surveys [54]. Soluble surface fractions of *H. pylori* were found to enhance the activity of γ-secretase and promote Aβ42 formation, inducing cognitive impairment [55]. Taxonomic levels of *Bacteroides* were significantly lower in AD patients than in controls [51], and anti-inflammatory effects conferred by *Bacteroides* might contribute to the anti-AD effects. While *Erysipelotrichaceae* essentially corresponds to the soy oil-based lipid emulsion, its role in AD is still unknown [54]. Species of *Desulfovibrio* can produce hydrogen sulfide (H_2_S), a gas with potentially genotoxic effects, by metabolizing dietary sulfites, sulfates, and sulfomucin. This genus is documented to have increased prevalence in patients with ulcerative colitis and other diseases involving colon inflammation, as well as in dextran sulfate sodium salt (DSS)-challenged mice [56]. In a prior study, the abundance of *Desulfovibrionaceae*, at the family level, increased in AD mice [57]. Gut *Desulfovibrionales* were altered by the herbal medicine LW-AFC, and the change was correlated with learning abilities in the senescence-accelerated mouse prone 8 (SAMR8) strain, another mouse model mimic to AD [58]. However, the precise mechanisms involving *Desulfovibrio* and *Erysipelotrichaceae* in this study beyond neuro-inflammation require further investigation.

Inflammation induced by gut dysbiosis is a pivotal part of the gut-brain axis. The GM was found to regulate the amyloid burden. This regulation occurred by directly altering amyloid metabolism and aggregation. The GM was also found to enhance systemic and gut inflammation, which increase microbial amyloid leakage from the gut to the brain [50]. In this study, the expression of Occludin and Claudin-5 in the jejunum reduced in AD mice. However, this reduction was reversed by PAW and FMT. These results supported the hypothesis that harmful microbiota can break down epithelial barrier integrity in AD [41]. PAW and FMT were found to alter GM profiles and rescued epithelial barrier integrity. This effect should prevent gut amyloid leakage. Neural inflammation plays an important role in AD pathophysiology [16]. The results of this study showed that inflammatory cytokines in the brain, including IL6, IL-1β, and TNF-α, were suppressed in both PAW-feeding and FMT AD mice. Although some inflammatory markers, including IL-1β and TNF-α, did not show a significant reduction in the cortex of PAW-feeding and FMT AD mice compared to water-feeding AD mice by ELISA, the IF assay showed a significant reduction in these markers. The total proteins collected from brain tissues might dilute the proportions of these inflammatory markers among total proteins, as inflammation likely only occurred in some restricted regions. Further analysis of neuroinflammation by M1 and M2 microglial markers showed that the ratio of Arginase-1/CD86 protein levels was reduced in AD mice compared to WT mice. However, this reduction was reversed in PAW-feeding and FMT mice compared to AD mice without treatment. These results support the hypothesis that the GM altered by PAW and FMT can have an anti-neuro-inflammation effect. Further analysis of the GM using the MetaCyc metabolic pathway showed prominent bacteria profiles in vitamin K synthesis. Vitamin K is suggested to upregulate AXL expression and then reduce neuroinflammation. In our present data, AXL expression in the brain was significantly reduced in AD mice compared with WT mice. However, this reduction was reversed in PAW-feeding and FMT AD mice. The activation of the AXL pathway might contribute to reducing neuroinflammation in PAW-feeding and FMT AD mice.

Both Aβ and tau play important roles in the pathophysiology of AD [42]. However, almost all clinical trials targeting the Aβ clearance failed [1]. The causes of the failure in those clinical trials were speculated mainly due to the late initiation of treatment. In this study, for establishing a stable GM, AD mice were treated with PAW for 1 month at 5 months of age. After that, the stool was collected and fed for 10 months to AD mice without PAW since 5 months old, when Aβ accumulation had not yet occurred. The results showed that FMT therapy diminished AD pathology and rescued cognitive declines. These findings support our hypothesis that PAW altered the GM and the changes in the GM improved AD through the gut-brain axis. Transplantation of fecal samples from a healthy volunteer or from an AD patient into germ-free animals produced stably colonized new GM and reproduced the bacterial profiles of the donors. Poor cognitive performance was detected in animals with fecal samples from AD subjects [59]. In a transgenic mouse model of AD, fecal microbiota from WT mice ameliorated the formation of amyloid plaques and neurofibrillary tangles, glial reactivity, and cognitive impairment [60]. All of these findings support GM being strongly linked to the pathophysiology of AD. However, this study is the first to prove that an altered fecal microbiota by specific water, PAW, can prevent transgenic AD mice from disease progression. It has been reported that female AD mice display more prominent AD symptoms than males [61]. The ovarian cycles are shown to be altered in a mouse model of AD, and the ovarian cycle stage contributes to AD-related network and cognitive impairments [62]. In this study, we used male AD mice for the study and found significant therapeutic effects of PAW. We hypothesized better results can be obtained by using female AD mice but it certainly needs further investigation. PAW, with intrinsic anti-oxidative and anti-inflammatory characteristics, can also change gut bacteria to achieve the same effect as effective FMT. Thus, delivering PAW could be a more natural, convenient, and promising way to treat AD.

## CONCLUSION

The GM profile was difference between AD and WT mice. PAW therapy altered GM profile, which was reproduced by FMT in AD mice. Inflammation, amyloid burdens, cognitive decline, neuroinflammation, and AD symptoms was also reduced by PAW therapy and FMT. The GM profile increased seen in PAW therapy and FMT showed the function on intestine integrity and anti-neuroinflammation in brain. PAW might be a potential therapy strategy in AD.

## Supplementary Materials

Supplementary Figure 1

Supplementary Tables
