# Supplementary material for: Plasmon-activated water as a therapeutic strategy in Alzheimer’s disease by altering gut microbiota
Source: Aging (Albany NY). 2023 May 8;15(9):3715–37. doi: 10.18632/aging.204706 (PMC10449311; doi:10.18632/aging.204706)
Supplement: Supplementary Figure 1 [file aging-15-204706-s001.pdf]

# SUPPLEMENTARY FIGURE

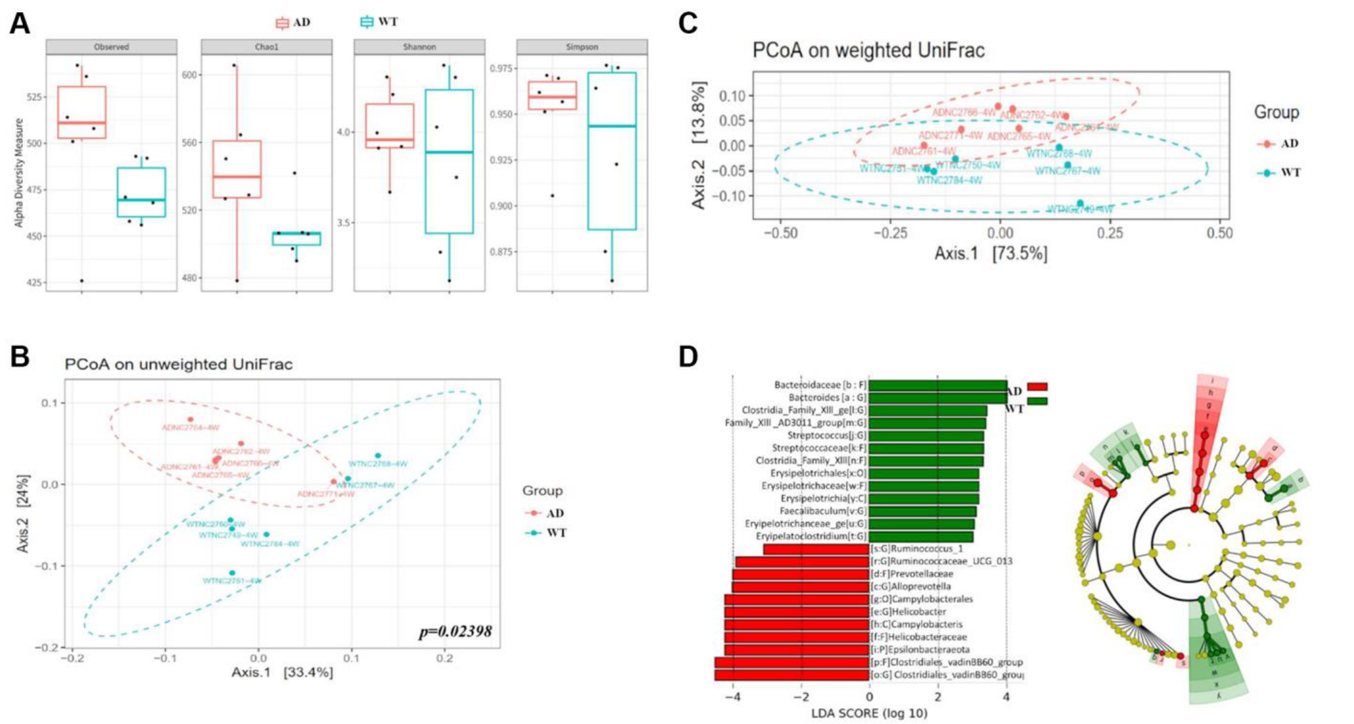


**Supplementary Figure 1. Fecal microbiome distribution analysis in wild-type (WT) and AD mice.** The feces of rats treated with and without plasmon-activated (PAW) was prepared for fecal microbiotic profiling by high-throughput sequencing of the 16s rRNA gene with the Illumina MiSeq system. (**A**) Alpha-diversity of PAW treated samples and untreated controls. Principal coordinate analysis (PCoA) plot based on (**B**) unweighted or (**C**) weight UniFrac distances of omeprazole treated samples and untreated controls. Significant differences in beta-diversity were evaluated with permutational multivariate analysis of variance (vegan::adonis, 1000 permutations) and beta-dispersion was quantified with betadisper (vegan::betadisper, 1000 permutations). Both indices achieved adonis $p < 0.05$ and betadisper $p > 0.05$. (**D**) Linear discriminant analysis (LDA) effect size (LEfSe) analysis of gut microbiotic changes in rats with long-term omeprazole treatment. Significant biomarkers were defined as taxa with an LDA score (log10) of ≥ 2. (b) Significant taxa were highlighted on the cladogram. Abbreviations: P: phylum; C: class; O: order; F: family; G: genus.
