# Supplementary material for: Plasmon-activated water as a therapeutic strategy in Alzheimer’s disease by altering gut microbiota
Source: Aging (Albany NY). 2023 May 8;15(9):3715–37. doi: 10.18632/aging.204706 (PMC10449311; doi:10.18632/aging.204706)
Supplement: Supplementary Tables [file aging-15-204706-s002.pdf]

SUPPLEMENTARY TABLES

Supplementary Table 1. Relative abundance (%) of gut microbiota with water (WT, AD) and PAW-fed (WT+PAW, AD+PAW) mice.

Genus	AD	AD+PAW	WT	WT+PAW
Desulfovibrionaceae_ge	0.0023% ± 0.0019%	2.9352% ± 4.6567%	0.0039% ± 0.0029%	0.2052% ± 0.3204%
Desulfovibrio	0.0499% ± 0.0438%	0.0079% ± 0.0038%	0.0225% ± 0.0133%	0.0242% ± 0.0284%
Erysipelatoclostridium	0.0143% ± 0.0288%	0.1179% ± 0.1370%	0.1262% ± 0.0959%	0.0499% ± 0.0505%
Candidatus_Stoquefichus	0.0107% ± 0.0161%	0.0690% ± 0.0681%	0.0064% ± 0.0067%	0.0220% ± 0.0252%
Allobaculum	0.0011% ± 0.0016%	1.8756% ± 2.3010%	0.0011% ± 0.0009%	0.1375% ± 0.1974%
Ruminococcus_1	0.1829% ± 0.1388%	1.2133% ± 1.8986%	0.0399% ± 0.0404%	0.0143% ± 0.0233%
Akkermansia	0.1055% ± 0.0767%	1.3044% ± 1.6261%	0.3007% ± 0.6173%	0.1414% ± 0.1392%
Mucispirillum	0.0392% ± 0.0651%	0.0273% ± 0.0649%	0.0047% ± 0.0056%	0.0007% ± 0.0012%
Lachnospiraceae_FCS020_group	0.1033% ± 0.1265%	0.0312% ± 0.0178%	0.1182% ± 0.0861%	0.3206% ± 0.2779%
Tyzzerella	0.0393% ± 0.0214%	0.0152% ± 0.0103%	0.0170% ± 0.0107%	0.0304% ± 0.0196%
Helicobacter	4.3627% ± 1.4373%	2.0233% ± 2.2687%	0.8982% ± 0.5357%	1.9475% ± 1.4251%

Supplementary Table 2. Relative abundance (%) Genus level of wildtype (WT) and AD mice gut microbiota with PAW treatment (WT+PAW, AD+PAW) and fecal microbiota transplantation (WT+FMT, AD+FMT).

Genus	AD+PAW	AD+FMT	WT+PAW	WT+FMT
Desulfovibrionaceae_ge	4.323%	3.287%	1.345%	2.070%
Desulfovibrio	0.016%	0.016%	0.018%	0.023%
Erysipelatoclostridium	0.004%	0.008%	0.002%	0.008%
Candidatus_Stoquefichus	0.012%	0.013%	0.020%	0.017%
Allobaculum	0.192%	0.073%	0.912%	0.087%
Ruminococcus_1	0.051%	0.101%	0.030%	0.131%
Akkermansia	0.383%	0.094%	0.155%	0.897%
Mucispirillum	0.227%	0.077%	0.240%	0.011%
Lachnospiraceae_FCS020_group	0.003%	0.001%	0.013%	0.002%
Tyzzerella	0.237%	0.089%	0.067%	0.043%
Helicobacter	0.834%	0.924%	0.395%	0.166%

Supplementary Table 3. The standardized uptake value ratio (SUVR) in the cerebellum of each brain region (Mean ± SEM) from wildtype (WT) and AD mice fed with water (WT, AD), PAW (WT+PAW, AD+PAW) and FMT (WT+FMT, AD+FMT).

Region	WT	WT+PAW	WT+FMT	AD	AD+PAW	AD+FMT
Cortex	0.960 ± 0.024	0.967 ± 0.024	0.955 ± 0.013	1.127 ± 0.046	1.044 ± 0.040	1.043 ± 0.045
Hippocampus	1.026 ± 0.021	1.030 ± 0.019	0.962 ± 0.020	1.175 ± 0.048	1.083 ± 0.031	1.024 ± 0.042
Striatum	0.988 ± 0.017	0.993 ± 0.017	1.044 ± 0.025	1.077 ± 0.029	1.045 ± 0.031	1.096 ± 0.036
Forebrain bundle	1.008 ± 0.017	1.005 ± 0.018	0.958 ± 0.023	1.147 ± 0.060	1.024 ± 0.028	1.029 ± 0.034

Supplementary Table 4. The protein concentration (pg/mg) of cytokines extracted from cortex and hippocampus of WT and AD fed with water (WT, AD), PAW (WT+PAW, AD+PAW) and FMT (WT+FMT, AD+FMT) mice were measured by enzyme-linked immunosorbent assay (ELISA) (Mean ± SEM).

Cytokine	Region	WT	WT+PAW	WT+FMT	AD	AD+PAW	AD+FMT
IL-6	Cortex	121 ± 9	150 ± 28	156 ± 27	133 ± 26	119 ± 28	110 ± 9
	Hippocampus	75 ± 11[a]	62 ± 25[a]	64 ± 4[a]	154 ± 56[b]	74 ± 8[a]	67 ± 8[a]
TNF-α	Cortex	238 ± 34	316 ± 61	354 ± 42	179 ± 79	320 ± 90	361 ± 68
	Hippocampus	652 ± 172[a]	87 ± 50[b]	130 ± 45[b]	688 ± 150[a]	99 ± 53[b]	194 ± 53[b]
IL-1β	Cortex	177 ± 25[a]	43 ± 6[b]	54 ± 10[b]	298 ± 46[c]	211 ± 21[a]	220 ± 41[a]
	Hippocampus	1471 ± 32[a]	783 ± 146[b]	813 ± 71[b]	1595 ± 147[a]	1110 ± 61[b]	1120 ± 104[b]

[a,b,c]Different superscripts within a row (between columns) indicate significant differences between groups ($p < 0.05$).
